# The uncharacterized *E. coli* gene *ybjD* encodes a novel Class 1 OLD protein

**DOI:** 10.17912/micropub.biology.002197

**Published:** 2026-05-28

**Authors:** Leslie T. Atahualpa, Jordan L. Polster, Jennifer Monzon, Bryan H. Thurtle-Schmidt

**Affiliations:** 1 Biology Department, Davidson College, Davidson, NC 28035, USA

## Abstract

Overcoming Lysogenization Defect (OLD) proteins demonstrate anti-phage defense in multi-gene systems including the Gabija system and some retrons, but isolated OLD genes remain poorly understood. Here we show the uncharacterized *E. coli* gene *ybjD* encodes an OLD protein producing anti-phage phenotypes upon induced expression. The Toprim domain is dispensable while the ATPase domain is essential for these phenotypes. Unexpectedly, a Walker A mutant predicted to prevent ATP binding retains anti-phage phenotypes but exhibits enhanced cytotoxicity. Together, these findings suggest ATP binding modulates YbjD activity. We propose renaming *ybjD* as *oldB* , a Class 1 OLD protein in *E. coli* .

**Figure 1. Molecular and phenotypic characterization of the E. coli OLD protein YbjD:** ( **A** ) Multiple sequence alignment of YbjD with established OLD proteins. The K35 within the Walker A motif of YbjD is highlighted in red. ( **B** ) Genomic context of *ybjD* in the *E. coli* genome. ( **C** ) RT-qPCR analysis using the Pfaffl method comparing *ybjD* expression in the Keio knockout strain and the parental strain. ( **D** ) Plaque assay results showing phage plating on lawns expressing YbjD under 0.001% arabinose induction; images are representative of at least three independent biological replicates. ( **E** ) Growth curve analysis measuring optical density over time for strains expressing wild-type or mutant YbjD under the indicated arabinose concentrations. Error bars correspond to standard deviations from three independent biological replicates. ( **F** ) Representative plaque assay showing plaque size reduction for phage Bas64 upon YbjD expression. ( **G** ) Cell killing assay in a temperature-sensitive *recBC* strain under repressive (glucose) or inducing (arabinose) conditions for P2 Old or YbjD expression.

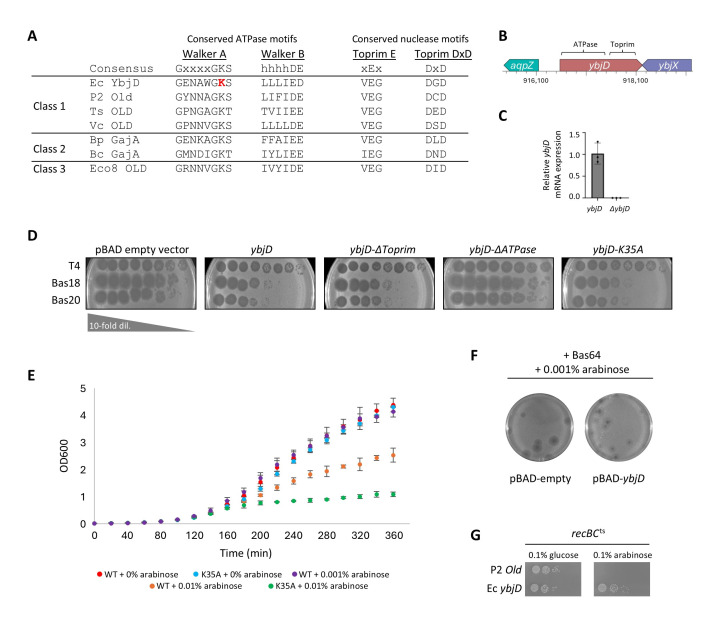



## Description

Bacteria have evolved numerous anti-phage defense systems, the most well-studied of which include CRISPR and restriction-modification systems. Genomic analyses continue to identify myriad other anti-phage defense systems, many of which remain poorly understood(Rodriguez-Rodriguez et al., 2025; DeWeirdt et al., 2026; Mordret et al., 2026). Some systems feature Overcoming Lysogenization Defect (OLD) proteins, which possess an N-terminal ATPase domain and a C-terminal Toprim domain and have been shown to mediate anti-phage defense in multi-gene systems such as the Gabija system or in a subset of retrons(Dot et al., 2023; Akritidou and Thurtle-Schmidt, 2023). In contrast to the widespread distribution of Class 2 OLD proteins in Gabija systems and Class 3 OLD proteins in retrons, isolated Class 1 OLD proteins are rare, occurring in only 0.45% of bacterial genomes(Patel and Seed, 2025).

We hypothesized that *E. coli* might encode unknown OLD proteins with the potential to provide anti-phage phenotypes on their own. We chose to investigate *ybjD* because its sequence identified it as a potential OLD protein ( **Fig. 1A** ). A recent analysis of the *E. coli* genome shows *ybjD* belongs to the 15.5% of protein-coding genes in *E. coli* that remain completely uncharacterized(Moore et al., 2024). To begin characterizing *ybjD* , we performed a multiple sequence alignment with previously studied OLD proteins P2 Old(Myung and Calendar, 1995), *V. cholerae* OLD(Patel and Seed, 2025), *T. scotoductus* OLD(Schiltz et al., 2020), *B pseudomallei* GajA(Schiltz et al., 2019), *B. cereus* GajA(Cheng et al., 2021), and the retron Eco8 OLD protein(Millman et al., 2020) ( **Fig. 1A** ). The alignment shows that YbjD possesses both the conserved Walker A and Walker B motifs in its N-terminal ATPase, as well as the conserved acidic amino acids that comprise the putative nuclease active site in the Toprim domain, suggesting that *ybjD* encodes an OLD protein.

The genomic context of *ybjD* within the *E. coli* genome identifies YbjD as a Class 1 OLD protein ( **Fig. 1B** ). Class 1 OLD proteins are distinguished by their isolated genomic context, as opposed to Class 2 and Class 3 OLD proteins, which are part of multi-gene anti-phage defense systems as in Gabija and certain retrons, respectively(Dot et al., 2023; Akritidou and Thurtle-Schmidt, 2023). The *ybjD* gene is flanked by a known aquaporin gene *aqpZ* on one side, while the other side is flanked by the gene *ybjX* (Herson et al., 2024), which is of unknown function(Moore et al., 2024). The *ybjD* and *ybjX* genes are encoded on opposing strands in a convergent orientation and therefore are unlikely to share a promoter or be part of a multi-gene system.

To test for any phenotypes associated with *ybjD* , we obtained a *ΔybjD* knockout from the Keio collection along with the parent strain(Baba et al., 2006). First, we determined whether *ybjD* is expressed in the parent strain and validated the knockout strain by RT-qPCR. Expression of *ybjD* was detected in the parent strain but not in the knockout strain ( **Fig. 1C** ). Plaque assays comparing the parent and *ΔybjD* strains showed no difference in phage susceptibility, so we tested whether complementation in the knockout *ΔybjD* strain with arabinose-inducible YbjD expression from a plasmid could reveal anti-phage phenotypes.

To test for anti-phage phenotypes, we performed plaque assays with all 68 members of the original BASEL phage collection(Maffei et al., 2021). Upon titrating arabinose we found that a concentration of 0.001% was sufficient to demonstrate anti-phage phenotypes but was not toxic to cells ( **Fig. 1D, 1E** ). Our data show that induced expression of YbjD reduces plaquing by the siphoviruses Bas18 and Bas20. The only other phenotype was observed against Bas64-Bas68, T7-related phages within the *Autographiviridae* family, where YbjD expression reduced plaque size without causing a fold-change reduction in plaque formation ( **Fig. 1F** ).

Initial attempts at mutagenesis of the three putative catalytic acidic amino acids in the Toprim domain yielded no phenotypic differences, so we decided to delete the entire Toprim domain to test whether it is dispensable for anti-phage phenotypes. Unexpectedly, the truncated ΔToprim construct retained anti-phage phenotypes indistinguishable from those of the full-length construct ( **Fig. 1D** ). In contrast, deleting the ATPase domain resulted in complete loss of anti-phage phenotypes. To test the role of ATP binding, we constructed a Walker A mutant, K35A. Strikingly, the K35A mutant retained anti-phage phenotypes in plaque assays ( **Fig. 1D** ). Additionally, bacterial cell lawns appeared thinner in plaque assays using the K35A construct, so we decided to test its cytotoxicity in growth assays. Uninduced controls grew normally, while cells expressing the wild-type protein showed some cytotoxicity at arabinose concentrations of 0.01%. Cells expressing the K35A mutant, however, showed significantly enhanced cytotoxicity ( **Fig. 1E** ). These results suggest that perturbation of ATP binding exacerbates YbjD cytotoxicity. Importantly, the anti-phage phenotypes were observed at 0.001% arabinose, a concentration at which we did not observe toxicity from expression.

We next decided to test whether the mechanism of cytotoxicity is the same as that of P2 Old, the archetypal Class 1 OLD protein. P2 Old is activated by sensing RecBC inhibition by the lambda protein gam(Sironi et al., 1971). Once activated, P2 Old cleaves a host tRNA(Govande et al., 2025). Our data show that the expression of P2 Old but not YbjD in a temperature-sensitive recBC- strain is lethal to cells ( **Fig. 1G** ). These results indicate YbjD employs a RecBC-independent mechanism of toxicity distinct from that of P2 Old.

Collectively, our data suggest that YbjD activity is influenced by ATP binding, and that perturbation of ATP binding increases cytotoxicity without abolishing anti-phage phenotypes. Nucleotide-dependent regulatory mechanisms have been observed in the GajA OLD protein in the *B. cereus* Gabija system, in which ATP binding inhibits DNA binding and cleavage(Cheng et al., 2023). In contrast, ATPase activity is required for anti-phage defense in other characterized OLD proteins such as Vc OLD, where mutation of conserved ATP-binding residues abolishes anti-phage defense(Patel and Seed, 2025). Together, these findings indicate that ATPase domains can play distinct roles across OLD proteins, ranging from regulatory modulation to essential catalytic functions.

Further studies will be required to define the physiological role of YbjD in *E. coli* . It is possible, for example, that YbjD harbors additional activities beyond the anti-phage phenotypes we show here. A recent study identified an archaeal OLD protein from *S. islandicus* named Cran1 as an essential gene involved in cell cycle progression(Yang et al., 2026). In contrast, YbjD is not essential in *E. coli* , but the observation that its Toprim domain is dispensable for the anti-phage phenotypes observed in this study suggests that the Toprim domain may function in contexts not yet identified. Despite these remaining uncertainties, based on its sequence homology, domain organization, genomic context, and anti-phage phenotypes, we propose renaming *ybjD* as *oldB* , a novel Class 1 OLD protein in *E. coli* .

## Methods

*Plasmid construction and mutagenesis*

The *ybjD* coding sequence (residues 1–552) was amplified from *E. coli* genomic DNA using primers CGTTTTTTGGGCTAACAGGAGGAATTAACCATGATTCTTGAGCGCGTTGAAATTGTGGG and ATCTTCTCTCATCCGCCAAAACAGCCATTAATCCGCGCGACCGCGCGCCAGCCACAG and cloned into a pBAD expression vector under control of an arabinose-inducible promoter using Gibson assembly. The ΔToprim construct encodes residues 1-377, and the ΔATPase construct encodes residues 378-552 cloned downstream of a start codon. The K35A Walker A mutant was generated by site-directed mutagenesis, and all constructs were verified by Sanger sequencing.

*RT-qPCR analysis of ybjD expression*

The relative *ybjD* mRNA expression in the Keio YbjD knockout and parent *E. coli* K-12 BW25113 strains was assessed via RT-qPCR. The parent strain genotype is: rrnB3 ΔlacZ4787 hsdR514 Δ(araBAD)567 Δ(rhaBAD)568 rph-1, while the knockout strain genotype is identical but with the *ybjd* gene replaced by a kanamycin resistance cassette. RNA was isolated using the Direct-zol RNA miniprep kit (Zymo Research). Samples were then prepared for RT-qPCR using the LunaUniversal One-Step RT-qPCR Kit (New England Biolabs). The primers used for *ybjD* were CGCGGTATCAACCGTTTGTC and AATCGTCGCGCTCAAAATGG. The primers used for the reference gene, *hcaT* , were GCTGCTCGGCTTTCTCATCC and CCAACCACGCTGACCAACC. Expression was quantified using three independent biological replicates and analyzed using the Pfaffl method, normalized to the reference gene *hcaT* (Zhou et al., 2011).

*Plaque assay for anti-phage phenotypes*

Overnight cultures of the appropriate strains were inoculated from freshly streaked plates and grown at 37°C in 5 mL LB supplemented with 100 μg/mL ampicillin and 0.05% glucose to repress pBAD-driven expression. The following day, cultures were diluted to an OD600 of 0.05 and grown in new 5 mL cultures with 100 μg/mL ampicillin and no glucose. Upon reaching an OD600 of 0.4, the cells were mixed with top agar at a ratio of 150 μL cells per 10 mL of top agar. Top agar was LB supplemented with 0.5% agar, 100 μg/mL ampicillin, 5 mM CaCl _2_ , 5 mM MgCl _2_ , 0.1 mM MnCl _2_ , and 0.001% arabinose. The top agar and cells were mixed and 5 mL were plated on LB plates containing 2% agar and 100 μg/mL ampicillin. Plates were incubated for 15 min at room temperature, followed by 30 minutes at 37°C to allow for arabinose induction and a final 15 min incubation with cracked lids in a fume hood to facilitate drying. Ten-fold dilutions of phage from a starting 10 ^9^ PFU/mL titer were prepared with phage dilution buffer (68 mM NaCl, 10 mM Tris pH 7.4, and 10 mM MgCl _2_ ) and 5 μL were plated. Plates were incubated overnight at 37°C and imaged the following day. Images shown are representative of at least three independent biological replicates. Phenotypes were assessed using the Keio Δ *ybjD* strain because this strain lacks the genomic *ybjD* locus, ensuring that observed phenotypes arise solely from plasmid-borne expression. Additionally, the Δ *araBAD* background prevents metabolism of arabinose, helping maintain a stable inducer concentration during the assay at low arabinose levels of 0.001%. For the plaque size assays against *Autographiviridae* , phage dilutions were premixed with cells prior to addition of top agar and plating.

*Cell growth cytotoxicity assays*

Strains with the indicated genotypes were grown in LB starter cultures supplemented with 100 μg/mL ampicillin and shaken at 37°C overnight. The following day, cultures were diluted to an OD600 of 0.02 in flasks containing LB supplemented with 100 μg/mL ampicillin and arabinose at final concentrations of 0%, 0.01%, or 0.001%. OD600 measurements were taken every 20 minutes for 6 hours. Error bars represent standard deviations from three independent biological replicates.

*Temperature-sensitive recBC assay*

A *recBC* temperature-sensitive *E. coli* strain (SK129)(Kushner, 1974) was transformed with 40 ng of the pBAD vector encoding either P2 Old or YbjD. Cells were incubated on ice for 30 min, heat shocked at 42°C for 30 s, and then recovered in 200 μL LB for 60 min at 30°C. Serial 5-fold dilutions of cells were prepared in LB, and 5 μL was plated onto LB agar plates containing 2% agar, 100 μg/mL ampicillin, and either 0.1% arabinose for induction or 0.1% glucose for repression. Plates were incubated overnight at 37°C and imaged the following day. Images shown are representative of three independent biological replicates.
